# Determinants of Stillbirth From Two Observational Studies Investigating Deliveries in Kano, Nigeria

**DOI:** 10.3389/fgwh.2021.788157

**Published:** 2022-01-14

**Authors:** Rebecca Milton, Fatima Zara Modibbo, William John Watkins, David Gillespie, Fatima Ibrahim Alkali, Murjanatu Bello, Chinagozi Precious Edwin, Fatima Habib Sa ad, Kerenza Hood, Kenneth Iregbu, Aishatu Kassim, Rashida Yakubu Khalid, Maryam Yahaya Muhammad, Aisha Sani Mukaddas, Ese Ogudo, Fatima Muhammad Tukur, Timothy Rutland Walsh

**Affiliations:** ^1^Centre for Trials Research, Cardiff University, Cardiff, United Kingdom; ^2^Murtala Muhammad Specialist Hospital, Kano, Nigeria; ^3^Department of Infection and Immunity, School of Medicine, Cardiff University, Cardiff, United Kingdom; ^4^Department of Microbiology, Aminu Kano Teaching Hospital, Kano, Nigeria; ^5^National Hospital Abuja, Abuja, Nigeria; ^6^Department of Zoology, Ineos Institute of Antimicrobial Research, University of Oxford, Oxford, United Kingdom

**Keywords:** stillbirth, stillborn, global health, neonatal, maternal, epidemiology, Nigeria, low- and middle-income countries

## Abstract

**Background:**

Stillbirths are a poignant representation of global inequality. Nigeria is documented to have the second highest rate; yet, the reporting system is inadequate in most Nigerian healthcare facilities. The aim was to identify the determinants of stillbirth among deliveries in the Murtala Muhammad Specialist Hospital (MMSH), Kano, Nigeria.

**Methods:**

Two study designs were used: a case-control study (S1) and a prospective cohort study (S2). Both studies were carried out at the MMSH. For S1, stillbirths were retrospectively matched to a livebirth by time (target of 24 hours' time variation) to establish a case-control study with a 1:1 ratio. Eligibility into S2 included all mothers who were presented at the MMSH in labour regardless of birth outcome. Both were based on recruitment durations, not sample sizes (3 months and 2 months, respectively, 2017–2018). The demographic and clinical data were collected through paper-based questionnaires. Univariable logistic regression was used. Multivariable logistic regression was used to explore relationships between area type and other specific factors.

**Findings:**

Stillbirth incidence in S2 was 180/1,000 births. Stillbirth was associated with the following factors; no maternal education, previous stillbirth(s), prematurity, living in both semi-rural and rural settings, and having extended time periods between rupture of membranes and delivery. Findings of the multivariable analysis (S1 and S2) indicated that the odds of stillbirth, for those living in a rural area, were further exacerbated in those mothers who had no education, lived in a shack, or had any maternal disease.

**Interpretation:**

This research identifies the gravity of this situation in this area and highlights the need for action. Further understanding of some of the findings and exploration into associations are required to inform intervention development.

**Funding:**

This collaboration was partially supported by funding from Health and Care Research Wales.

## Introduction

Stillbirths are one of the most neglected tragedies in global health today; ~2.6 million stillbirths occur each year with 98% occurring in low- and middle-income countries (LMICs) (1–3) and Nigeria is reported to account for 12% of the 2.6 million (4). Around half of the stillbirths occur intrapartum and are commonly due to preventable conditions (5, 6).

At present, it is reported that it would take 160 years before a pregnant woman in Africa has the same opportunity of having a livebirth as a woman in a high-income country (HIC) (6). Average rates of stillbirth in LMICs are 25/1,000 births; 10-times higher than in HICs, and it is reported that the majority (57%) of stillbirths in LMICs are in rural settings (7–9).

Globally, 10 countries carry >65% of the burden of stillbirths, with Nigeria ranked second to Pakistan (10, 11). In a recent UNICEF report, a stillbirth rate of 43/1,000 was documented for Nigeria (12). Other studies in Nigeria carried out between 2008 and 2012 show rates ranging from 22.4 to 127/1,000 births (13). The United Nations' Every Newborn Action Plan has set a goal of 12/1,000 births by 2030 for all countries (7, 14). An underestimation of stillbirths in LMICs is not uncommon and often attributed to the absence of a functional stillbirth register along with other factors such as unregistered community births (15). At the Murtala Muhammad Specialist Hospital (MMSH) stillbirths were not registered, masking the magnitude of this tragedy. There is a paucity of data on determinants of stillbirths from LMICs, particularly from sub-Saharan Africa. The MMSH was a partner in a multi-site international research project entitled “Burden of Antibiotic Resistance in Neonates from Developing Societies” (BARNARDS) (www.barnards-group.com). The MMSH research team highlighted the high number of stillbirths delivered throughout BARNARDS, within which stillbirth incidence was not recorded. The aim of this study was to investigate the determinants of stillbirths among deliveries in the MMSH, Kano, Nigeria. To allow for international comparison, this study has used the WHO definition of stillbirth; a baby born with no signs of life at or after 28 weeks gestation (3).

## Methods

### Setting

The MMSH is a tertiary hospital located in Kano, northern Nigeria, and the population this hospital serves is ~11 million. Within the MMSH, there are 1,000–1,200 general hospital beds, 17 neonatal intensive care unit (NICU) beds, 133 maternity beds, and 22 delivery cubicles. Each month, there are around 550 deliveries and there are four midwives on shift at any one time, two for complicated deliveries and two for uncomplicated deliveries.

### Study Design, Ethical Approval, Informed Consent and Procedures

Data were collected through two exploratory studies and ethical approval for both studies was obtained locally. Study 1 (S1), a retrospective case-control study, was conducted without funding; to investigate associations and to understand the complexities surrounding data collection. Data and experiences from S1 informed the design of Study 2 (S2); a prospective cohort study, whereby incidence could be determined.

The S1 (case-control) stillbirth data were collected between 13/09/2017 and 31/12/2017. Trained research assistants were present at all deliveries and if the neonate was confirmed stillborn by a medical professional, the mother was approached to participate in this study, and a pre-tested paper-based questionnaire was completed after obtaining informed consent. Stillbirths were retrospectively matched to a livebirth from BARNARDS by time (target of 24 h time variation) to establish a case-control study with a 1:1 ratio. The maximum time between stillbirth and matched livebirth was 29 days, due to BARNARDS ceasing enrolment in early December 2017. BARNARDS procedures^1^ were followed to enrol controls. Sufficient relevant data were collected within BARNARDS to ensure that matched controls need not repeat the questionnaires.

The S2 (prospective cohort study) data were collected between 06/03/2018 and 22/04/2018 from all mothers presenting to MMSH while in labour, with the outcome being live or stillbirth. The mothers were enrolled during labour after informed consent was obtained, an initial pre-tested paper-based questionnaire was completed during labour, and another after delivery, dependent on delivery outcome.

### Variables, Data Sources, and Measurement

The questionnaires captured the participant's information in three domains: maternal, household, and neonatal/birth. Clinical notes were not exclusively used for data collection; however, they were used for some data points (e.g., gestational status). The domains were selected to provide a holistic approach to the mother's characteristics, demographics, health background, and delivery, and they are linked to unaddressed issues of three delays in the Kano state: (i) recognition of danger signs and associated decisions to seek appropriate healthcare and knowledge of how to seek healthcare; (ii) access to healthcare (distance, cost, security [concern after usual hours], and transport cost; (iii) lack of adequate resources and equipment and supplies.

The household income was defined as extremely low, very low, low, and average, based on local expertise and knowledge. The average monthly income for Kano is US$250.

### Statistical Methods

Sample size calculations were not performed for either study, due to non-existent benchmark data. Both studies were based on recruitment duration rather than sample sizes. The intention of this study was to provide preliminary data to inform future work.

Descriptive data are presented both overall and by outcome (livebirth/stillbirth) as frequencies and percentages. For each variable of interest, a univariable logistic regression was used with stillbirth as the outcome. Conditional logistic regression was used for the case-control study, to account for matching. Results are presented as odds ratios (OR), 95% CI, and *p*-values and are described within their corresponding domain. Additionally, relationships between area type (urban, semi-rural, and rural) and the other variables were investigated through multivariable logistic regressions in two ways. First, the area type and each of the other variables, in turn, were included as main effects to identify the impact of adjusting for the other. Second, only interaction terms were included to identify differential effects. We examined differences in model coefficients without and after accounting for multiple births within the mothers and based on negligible differences chose to not account for these. Statistical analyses were conducted using Statistical Package for the Social Sciences (SPSS) Version 25 and R Version 3.5.1.

### Role of the Funding Source

The funder had no role in study design; in the collection, analysis, and interpretation of data; in the writing of the manuscript; nor in the decision to submit the paper for publication. The corresponding author confirms that she had full access to all the data in the study and had final responsibility for the decision to submit for publication.

## Results

### S1: Case-Control Study; Numbers Enrolled and Descriptive Analysis

**Figure 1:**
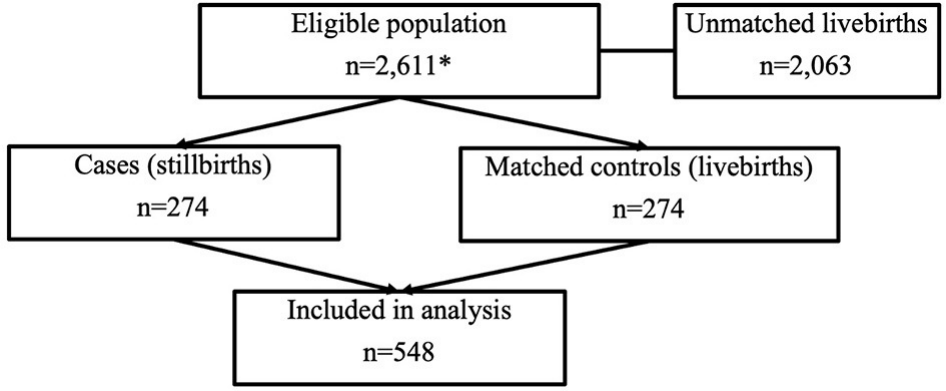
STROBE (16) flow diagram for case-control study. *Within the eligible population box, this details the total number of live and stillbirths throughout the time match period.

A total of 548 mothers; 274 stillbirths and 274 livebirths were included, as shown in Figure 1. Cases are defined as stillbirths and controls as matched livebirths. Data collection for controls ceased in December 2017, so cases born in this period were matched with controls born in late November 2017.

Of the mothers, 12% (*n* = 67) were <20 years old and 5% (*n* = 29) were ≥40 years, 20% (*n* = 108) were primigravida. The mothers who reported “any disease” accounted for 60% (*n* = 329), and 40% (*n* = 219) reported malaria. Over half (53% *n* = 291) of the mothers reported a minimum education level as secondary school/university, 26% (*n* = 142) reported limited schooling, and 21% (*n* = 115) reported no education. The mothers living in extremely low-income households represented 47% (*n* = 259), 46·4% (*n* = 254) reported living in low/very low, with only 6% (*n* = 35) living at average or above household income. Those without access water (defined as anything other than municipal network) represented 69% (*n* = 380), and 26% (*n* = 144) reported to live in shacks. Most were from urban (53%, *n* = 288) or semi-rural settings (37%, *n* = 203) with 10% (*n* = 57) residing in a rural setting. Observing deliveries, 77% (*n* = 424) were full-term (37–42 weeks), 13% (*n* = 69) were pre-term (<37 weeks) (17), and 10% (*n* = 55) were post-term >42 weeks) (18). There was no variation in gender distribution; 55.8% (*n* = 153) of stillbirths were male and 56.6% (*n* = 155) of livebirths were male (Table 1).

**Table 1:** Univariable results from S1: Case-control study detailing demographic characteristics, the distribution of variables within each of the domains, and the extent to which each variable is associated with stillbirth.

**Domain**	**Variable**	**Category**	**Overall (% in variable)**	**Livebirth (% in category)**	**Stillbirth (% in category)**	**OR**	**Lower 95% CI**	**Upper 95% CI**	***P*-value**
Maternal *n =* 548	Age band (years)	<20	67 (12.2)	23 (8.4)	44 (16.1)	2.385	1.327	4.289	0.004
		20–24	173 (31.6)	96 (35.0)	77 (28.1)	Reference category			
		25–29	120 (21.9)	65 (23.7)	56 (20.4)	1.074	0.674	1.713	0.764
		30–34	89 (16.2)	42 (15.3)	46 (16.8)	1.366	0.816	2.285	0.236
		35–39	70 (12.8)	33 (12.0)	37 (13.5)	1.398	0.801	2.440	0.239
		40+	29 (5.3)	15 (5.5)	14 (5.1)	1.164	0.529	2.558	0.706
	Education level	None	115 (21.0)	31 (11.3)	84 (30.7)	3.917	2.439	6.289	<0.001
		Limited schooling	142 (25.9)	71 (25.9)	71 (25.9)	1.445	0.966	2.164	0.074
		Secondary/University	291 (53.1)	172 (62.8)	119 (43.4)	Reference category			
	First pregnancy	Yes	108 (19.7)	69 (25.2)	39 (14.2)	0.494	0.320	0.764	0.002
		No—had previous livebirths	433 (79.0)	202 (73.7)	231 (84.3)	Reference category			
		No—previous pregnancy did not result in livebirth	7 (1.3)	3 (1.1)	4 (1.5)	1.166	0.258	5.272	0.842
	Previous stillbirth(s)	No	420 (76.6)	235 (85.8)	185 (67.5)	Reference category			
		Yes	128 (23.4)	39 (14.2)	89 (32.5)	2.899	1.899	4.424	<0.001
	Any disease	No	219 (40.0)	145 (52.9)	74 (27.0)	Reference category			
		Yes	329 (60.0)	129 (47.1)	200 (73.0)	3.038	2.126	4.341	<0.001
	Malaria	No	326 (59.5)	161 (58.8)	165 (60.2)	Reference category			
		Yes	222 (40.5)	113 (41.2)	109 (39.8)	0.941	0.669	1.324	0.728
	Hypertension	No	495 (90.3)	262 (95.6)	233 (85.0)	Reference category			
		Yes	53 (9.7)	12 (4.4)	41 (15.0)	3.842	1.972	7.486	0.001
	Infection/Fever	No	531 (96.9)	270 (98.5)	261 (95.3)	Reference category			
		Yes	17 (3.1)	4 (1.5)	13 (4.7)	3.362	1.082	10.445	0.036
	Other health condition	No	446 (81.4)	254 (92.7)	192 (70.1)	Reference category			
		Yes	102 (18.6)	20 (7.3)	82 (29.9)	5.424	3.213	9.155	<0.001
Household *n =* 548	Income^*^	Extremely low	259 (47.3)	151 (55.1)	108 (39.4)	Reference category			
		Very low	115 (21.0)	63 (23.0)	52 (18.9)	1.154	0.741	1.796	0.526
		Low	139 (25.4)	55 (20.1)	84 (30.7)	2.110	1.385	3.214	<0.001
		Average	35 (6.4)	5 (1.8)	30 (11.0)	8.669	3.265	23.012	<0.001
	Type of area	Urban	288 (52.6)	177 (64.6)	111 (40.5)	Reference category			
		Semi-Rural	203 (37)	85 (31.0)	118 (43.1)	2.214	1.535	3.193	<0.001
		Rural	57 (10.4)	12 (4.48)	45 (16.4)	5.980	3.031	11.798	<0.001
	Type of house	House	204 (37.2)	111 (40.5)	93 (34.0)	Reference category			
		Apartment	197 (35.9)	100 (36.5)	97 (35.4)	1.158	0.782	1.714	0.464
		Shack	144 (26.3)	63 (23.0)	81 (29.6)	1.535	0.999	2.357	0.051
		Other	3 (0.5)	0 (0.0)	3 (1.1)	NA	NA	NA	NA
	Access to clean water	Yes	168 (30.7)	30 (11.0)	138 (50.4)	Reference category			
		No	380 (69.3)	244 (89.1)	136 (49.6)	0.121	0.078	0.190	<0.001
Neonatal/Birth *n =* 548	Gender (*n =* 548)	Male	308 (56.2)	155 (56.6)	153 (55.8)	0.971	0.693	1.361	0.863
		Female	240 (43.8)	119 (43.4)	121 (44.2)	Reference category			
	Gestation status (*n =* 548)	Premature	69 (12.6)	7 (2.6)	62 (22.6)	11.891	5.317	26.593	<0.001
		Late	55 (10.0)	24 (8.8)	31 (11.3)	1.734	0.984	3.056	0.057
		Full term	424 (77.4)	243 (88.7)	181 (66.1)	Reference category			
	Number of hours after ruptured membrane to delivery (*n =* 399)	≤ 1 h	218 (54.6)	142 (67.9)	76 (40.0)	Reference category			
		>1 to ≤ 4 h	47 (11.8)	26 (12.4)	21 (11.1)	1.509	0.797	2.859	0.207
		>4 to ≤ 12 h	38 (9.5)	9 (4.3)	29 (15.3)	6.021	2.710	13.373	<0.001
		>12 to ≤ 24 h	43 (10.8)	20 (9.6)	23 (12.1)	2.149	1.110	4.161	0.023
		>24 to ≤ 72 h	53 (13.3)	12 (5.7)	41 (21.6)	6.384	3.167	12.869	<0.001
	Singleton/Multiple	Singleton	525 (95.8)	257 (93.8)	268 (97.8)	Reference category			
		Multiple	23 (4.2)	17 (6.2)	6 (2.2)	0.338	0.131	0.872	0.025

* *Monthly household income classifications: Extremely low, < $50; Very low, $50-99; Low, $100-249; and Average, >=$250*.

*NB that within this type of study the percentages for comparison are calculated within case and control separately*.

### Associations With Stillbirth—Univariable Analysis

Within the maternal domain, associations with higher odds of stillbirth were identified among younger mothers (<20 years old) (OR: 2.39 95% CI: 1.32–4.29 *p* = 0.004) compared with those aged 20–24 years old, no formal education, when compared with secondary education (OR: 3.92 95% CI: 2.44–6.29 *p* < 0.001), and among the mothers who had experienced the previous stillbirth(s) (OR: 2.90 95% CI: 1.90–4.43 *p* < 0.001) compared with no previous stillbirth. The mothers who presented with “any disease” were three times more likely to deliver a stillbirth (OR: 3.04 95% CI: 2.13–4.34 *p* < 0.001) compared with those with no reported disease. On exploring specific maternal health conditions, hypertension (inclusive of pre-eclampsia) was the only condition that presented higher odds of stillbirth when compared with those without hypertension (OR: 3.84 95% CI: 1.97–7.49 *p* < 0.001) (Table 1).

The findings within the household domain associated with higher odds of stillbirth included a household income of average or above and a low household income both compared with an extremely low household income [(average or above: OR: 8.67 95% CI: 3.27–23.01 *p* < 0.001), (low income: OR: 2.11 95% CI: 1.86–3.21 *p* < 0.001)]. Those residing in semi-rural or rural locations had higher odds of stillbirth compared with an urban area [(semi-rural: OR: 2.21 95% CI: 1.54–3.19 *p* < 0.001) (rural: OR: 5.98 95% CI: 3.03–11.80 *p* < 0.001)] (Table 1).

Pre-term delivery compared with term delivery was associated with higher odds of stillbirth (OR: 11.9 95% CI: 5.31–26.59 *p* < 0.001) and when comparing membranes rupturing ≤1 h before delivery anything over 4 h prior to delivery was associated with a higher odds of stillbirth [(>4 to ≤12 h OR: 6.02 95% CI: 2.71–13.37 *p* < 0.001) (>12 to ≤24 h OR: 2.15 95% CI: 1.11–4.16 *p* = 0.023) (>24 h OR: 6.39 95% CI: 3.17–12.87 *p* < 0.001)] (Table 1).

### Associations With Stillbirth—Multivariable Analysis

The area type was associated with stillbirth independent of the following confounding variables: access to clean water, maternal education level, house type, maternal disease, previous stillbirth, and household income (Supplementary Tables 1–6).

**Figure 2:**
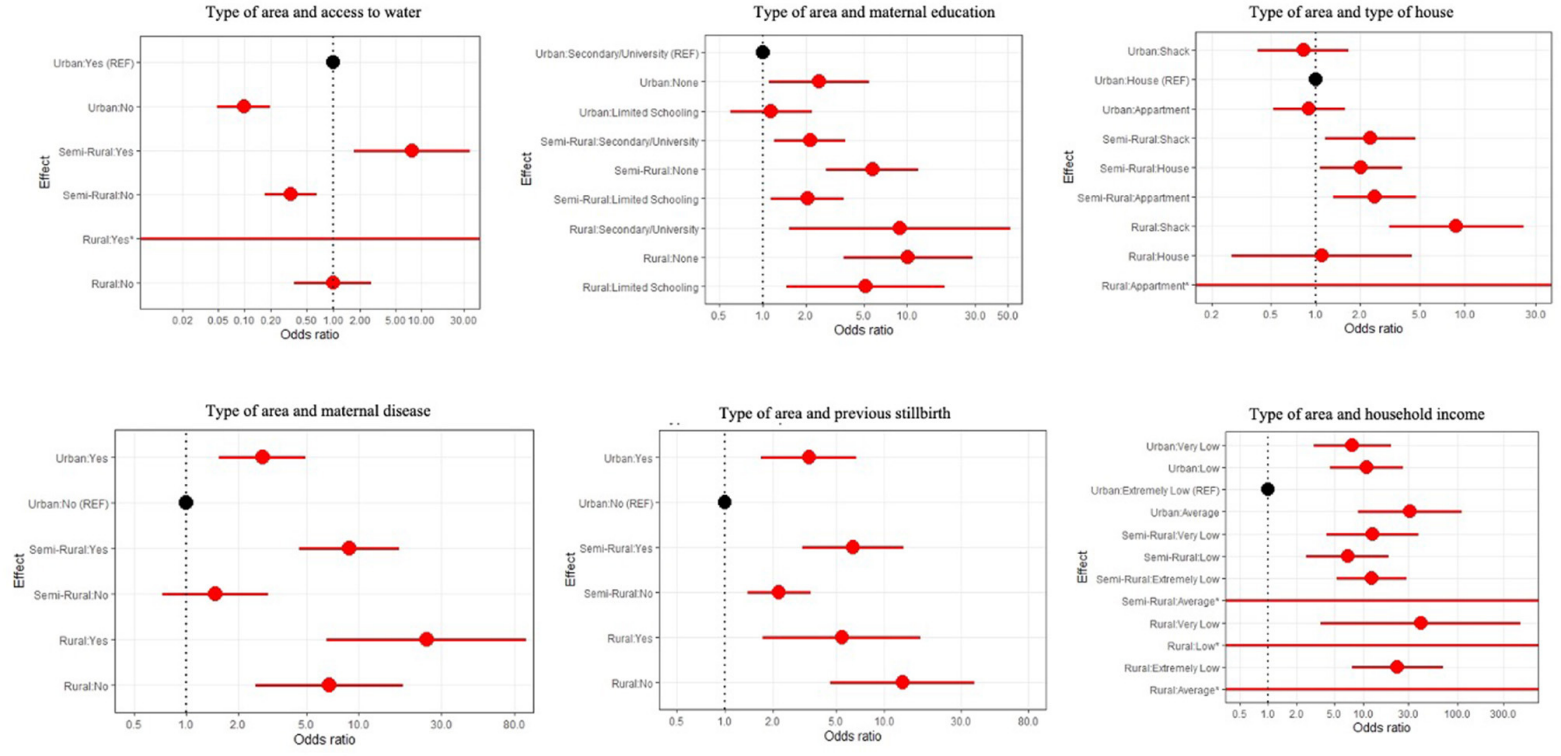
Forest plots for area type and the following confounding variables: access to clean water, maternal education, type of house, maternal disease, previous stillbirth(s), and household income.

The odds of stillbirth for the mothers living in a semi-rural area with access to clean water were seven times higher than the mothers in an urban area with access to clean water (OR: 7.78 95% CI: 1.73–34.91 *p* = 0.007) as shown in Figure 2. The interaction between education level and area type indicated that the mothers with no formal education had the highest odds of a stillbirth and this association was amplified for those from rural settings compared with those in an urban environment with secondary school level education (OR: 10.20 95% CI: 3.65–28.55 *p* < 0.001) (Figure 2). The odds of stillbirth were higher for rural mothers living in a shack compared with the mothers living in an urban area in a house (OR: 8.84 9% CI: 3.14–24.87 *p* < 0.001) (Figure 2). Living in a rural area with disease(s) was associated with higher odds of stillbirth when compared with those living in an urban area without a disease (Figure 2) (OR: 24.76 95% CI: 6.54–93.73 *p* < 0.001). In semi-rural and urban areas, previous stillbirth(s) was the largest predictor of subsequent stillbirth. Yet, in rural areas, mothers who had previously experienced a stillbirth(s) and lived in a rural area were over five times more likely to deliver a stillborn baby compared with mothers living in an urban setting and not having had a previous stillbirth (OR: 5.39 95% CI: 1.73–16.88 *p* = 0.004). Mothers who had not previously had a stillbirth but reported living rurally had higher odds again compared to mothers living in an urban setting and having not had a previous stillbirth (OR: 12.99 95% CI: 4.59–36.71 *p* < 0.001) (Figure 2). The interaction among area type, household income, and stillbirth found the mothers from rural areas with very low household income were 40 times more likely to deliver a stillborn baby than the mothers living in an urban setting with extremely low income (OR: 40.44 95% CI: 3.61–453.67 *p* = 0.003) (Supplementary Table 6).

### S2: Prospective Cohort Study; Numbers Enrolled, Descriptive Analysis, and Incidence of Stillbirth

**Figure 3:**
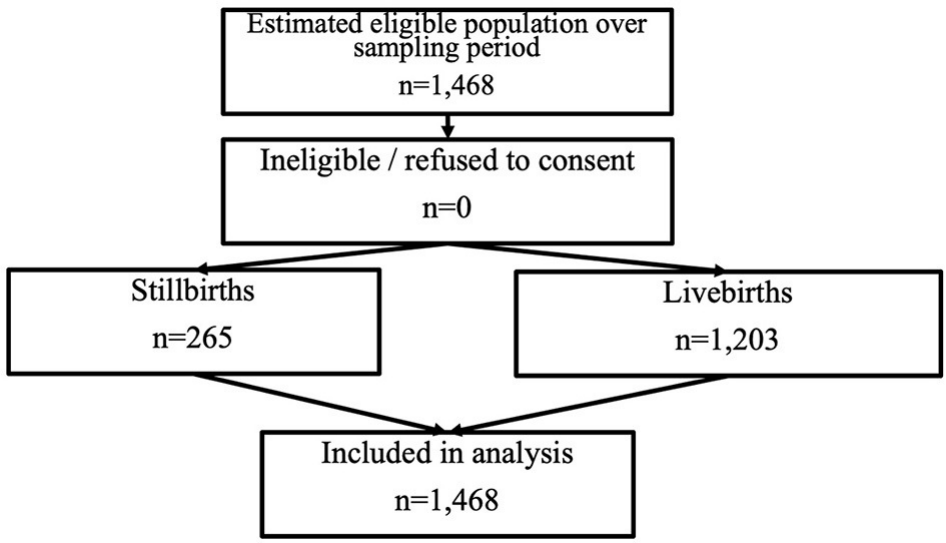
STROBE (16) flow diagram for prospective cohort study.

Within S2, 1,468 births were enrolled, comprising of 265 stillbirths and 1,203 livebirths, as shown in Figure 3. The stillbirth incidence was 180/1,000 births. The mothers aged <20 years represented 23% (*n* = 334) of the cohort, 6% ≥40 years (*n* = 85), 21% (*n* = 306) of the mothers were primigravida. The majority reported having “any disease” 62% (*n* = 916), with 20% (*n* = 294) reported having malaria. Those with no formal education made up 20% (*n* = 296), 21% (*n* = 304) had limited schooling, and 59% (*n* = 868) were educated to secondary school/university level. With 10% (*n* = 141) living in a household with average or above income, 45% (*n* = 663) did not have access to clean water, and 26% (*n* = 376) live in shacks. The majority lived in urban 53% (*n* = 781) and semi-rural settings; 38%, (*n* = 559) with only 9% (*n* = 128) in rural settings. This study included 788 male neonates; 139 stillbirths, and 678 female neonates; 124 stillbirths, the sex of two stillbirths were undetermined. The majority, 77% (*n* = 1,137), were delivered at term, 6% (*n* = 93) pre-term, and 13% (*n* = 238) post-term (Table 2).

**Table 2:** Univariable results from S2: Cohort study. Detailing demographic characteristics of the study participants, the distribution variables within each of the domains, and the extent to which each variable is associated with stillbirth.

**Domain**	**Variable**	**Category**	**Overall (% in variable)**	**Control (live born) (% in category)**	**Case (stillborn) (% in category)**	**OR**	**Lower 95% CI**	**Upper 95% CI**	***P*-value**
Maternal *n =* 1,468	Maternal age band	<20	334 (22.8)	288 (86.2)	46 (13.8)	0.794	0.530	1.190	0.264
		20–24	412 (28.1)	343 (83.3)	69 (16.8)	Reference category			
		25–29	339 (23.1)	272 (80.2)	67 (19.8)	1.224	0.844	1.776	0.286
		30–34	190 (12.9)	159 (83.7)	31 (16.3)	0.969	0.610	1.541	0.895
		35–39	108 (7.4)	79 (73.2)	29 (26.9)	1.825	1.109	3.002	0.018
		40+	85 (5.8)	62 (72.9)	23 (27.1)	1.844	1.070	3.177	0.027
	Maternal education level	None	296 (20.2)	206 (69.6)	90 (30.4)	2.646	1.936	3.617	<0.001
		Limited schooling	304 (20.7)	252 (82.9)	52 (17.1)	1.250	0.877	1.781	0.217
		Secondary/University	868 (59.1)	745 (85.8)	123 (14.2)	Reference category			
	Mothers first pregnancy	Yes	306 (20.8)	257 (84.0)	49 (16.0)	0.856	0.609	1.203	0.371
		No—had previous livebirths	1,142 (77.8)	934 (81.8)	208 (18.2)	Reference category			
		No—previous pregnancy did not result in livebirth	20 (1.4)	12 (60.0)	8 (40.0)	2.994	1.208	7.416	0.018
	Any previous stillbirths	Yes	1.229 (83.7)	159 (66.5)	80 (33.5)	2.839	2.081	3.875	<0.001
		No	239 (16·3)	1044 (85.0)	185 (15.1)	Reference category			
	Mother any disease	No	916 (62.4)	778 (84.9)	138 (15.1)	Reference category			
		Yes	552 (37.6)	425 (77.0)	127 (23.0)	1.685	1.288	2.203	0.001
	Malaria	No	1,174 (80.0)	962 (81.9)	212 (18.1)	Reference category			
		Yes	294 (20.0)	241 (82.0)	53 (18.0)	0.998	0.716	1.392	0.990
	Hypertension	No	1,307 (89.0)	1,084 (82.9)	223 (17.1)	Reference category			
		Yes	161 (11.0)	119 (73.9)	42 (26.1)	1.716	1.173	2.509	0.005
	Infection/Fever	No	1,420 (96.7)	1,168 (82.3)	252 (17.7)	Reference category			
		Yes	48 (3.3)	35 (72.9)	13 (27.1)	1.722	0.898	3.301	0.102
	Other condition	No	1,331 (90.7)	1,106 (83.1)	225 (16.9)	Reference category			
		Yes	137 (9.3)	97 (70.8)	40 (29.2)	2.027	1.365	3.010	<0.001
	Smoking history	Non-smoker	1,370 (93.3)	1,126 (82.2)	244 (17.8)	Reference category			
		Smoker	98 (6.7)	77 (78.6)	21 (21.4)	1.259	0.762	2.079	0.369
	Any substance abuse history?	No	1,385 (94.3)	1,134 (81.9)	251 (18.1)	Reference category			
		Yes	83 (5.7)	69 (83.1)	14 (16.9)	0.917	0.508	1.655	0.773
Household *n =* 1,468	Income^*^	Extremely low	217 (14.8)	164 (75.6)	53 (24.4)	Reference category			
		Very low	504 (34.3)	421 (83.5)	83 (16.5)	0.610	0.413	0.900	0.013
		Low	606 (41.3)	490 (80.9)	116 (19.1)	0.733	0.506	1.060	0.099
		Average	141 (9.6)	128 (90.8)	13 (9.2)	0.314	0.164	0.601	0.001
	Type of area	Urban	781 (53.2)	675 (86.4)	106 (13.6)	Reference category			
		Semi-Rural	559 (38.1)	442 (79.1)	117 (20.9)	1.686	1.263	2.250	<0.001
		Rural	128 (8.7)	86 (67.2)	42 (32.8)	3.110	2.039	4.743	<0.001
	Type of house	House	535 (36.4)	457 (85.4)	78 (14.6)	Reference category			
		Apartment	557 (37.9)	464 (83.3)	93 (16.7)	1.174	0.846	1.629	0.336
		Shack	376 (25.6)	282 (75.0)	94 (25.0)	1.953	1.397	2.730	0.001
	Access to clean water	Yes	805 (54.8)	676 (84.0)	129 (16.0)	Reference category			
		No	663 (45.2)	527 (79.5)	136 (20.5)	1.352	1.036	1.765	0.026
	Time to hospital	<1 h	1,171 (79.8)	979 (83.6)	192 (16.4)	Reference category			
		1–2 h	231 (15.7)	176 (76.2)	55 (23.8)	1.593	1.134	2.239	0.007
		More than 2 h	66 (4.5)	48 (72.7)	18 (27.3)	1.912	1.089	3.359	0.024
Neonatal/Birth *n =* 1,468	Gender	Male	788 (53.7)	649 (82.4)	139 (17.6)	0.957	0.732	1.250	0.747
		Female	678 (46.2)	554 (81.7)	124 (18.3)	Reference category			
		Undetermined	2 (0.1)	0 (0.0)	2 (100.0)	NA	NA	NA	NA
	Gestational status	Pre-term	93 (6.3)	40 (43.0)	53 (57.0)	7.383	4.749	11.478	<0.001
		Post term	238 (16.2)	199 (83.6)	39 (16.4)	1.092	0.747	1.596	0.649
		Full term	1,137 (77.5)	964 (84.8)	173 (15.2)	Reference category			
	Type or ward	Maternity departments	1,428 (97.3)	1,174 (82.2)	254 (17.8)	Reference category			
		Obstetrics and gynecology units	40 (2.7)	29 (72.5)	11 (27.5)	1.546	0.923	2.589	0.120
	How many hours after membranes ruptured to delivery	≤ 1 h	590 (40.2)	513 (87.0)	77 (13.1)	Reference category			
		>1 to ≤ 4 h	126 (8.6)	110 (87.3)	16 (12.7)	0.969	0.544	1.725	0.915
		>4 to ≤ 12 h	114 (7.8)	82 (71.9)	32 (28.1)	2.600	1.619	4.175	<0.001
		>12 to ≤ 24 h	105 (7.2)	86 (81.9)	19 (18.1)	1.472	0.848	2.555	0.170
		>24 to ≤ 72 h	116 (7.9)	95 (81.9)	21 (18.1)	1.473	0.867	2.502	0.152
		Unknown	417 (28.4)	317 (76.0)	100 (24.0)	NA	NA	NA	NA
	Singleton/Multiple	Singleton	1,325 (90.3)	1,074 (81.0)	251 (18.9)	Reference category			
		Multiple	143 (9.7)	129 (90.2)	14 (9.8)	0.464	0.263	0.819	0.008

* *Monthly household income classifications: Extremely low, < $50; Very low, $50-99; Low, $100-249; and Average, >=$250*.

### Associations With Stillbirth—Univariable Analysis

Higher odds of stillbirth within the maternal domain were found among the mothers aged ≥35 years, compared with the mothers aged between 20 and 24 years; (35–39 OR: 1.83 95% CI: 1.11–3.00 *p* = 0.018) (≥40 OR: 1.84 95% CI: 1.07–3.18 *p* = 0.027), and the mothers with no formal education compared with those with secondary school/university level (OR: 2.65 95% CI:1.94–3.62 *p* < 0.001). Mothers who have previously been pregnant, yet pregnancy did not result in a livebirth, had higher odds of stillbirth compared with mothers who had had a livebirth (OR: 2.99 95% CI: 1.21–7.42 *p* = 0.018). Previous stillbirth(s) was associated with higher odds of subsequent stillbirth (OR: 2.84 95% CI: 2.08–3.88 *p* < 0.001) when compared with no previous stillbirth. The mothers who reported having “any disease” had higher odds of stillbirth (OR: 1.69 95% CI: 1.23–2.20 *p* < 0.001) when compared with those with no reported disease. Hypertension was the only medical condition that presented with higher odds of stillbirth when compared with those without hypertension (OR: 1.72 95% CI: 1.17–2.51 *p* = 0.005) (Table 2).

Within the household domain, findings associated with a higher odds of stillbirth included living in either semi-rural or rural settings compared to living in an urban setting (semi-rural OR: 1.69 95% CI: 1.26–2.25 *p* < 0.001) (rural OR: 3.11 95% CI: 2.04–4.74 *p* < 0.001), living in a shack compared to a house (OR:1.95 95% CI:1.40–2.73 *p* < 0.001). Having no access to clean water, compared to having access to clean water had higher odds of stillbirth (OR: 1.35 95% CI: 1.04–1.77 *p* = 0.026). Having an extended travel time to the hospital was associated with higher odds of stillbirth; compared with <1 h journey time. Any journey over 1 h had higher odds (1–2 h OR: 1.59 95% CI: 1.13–2.24 *p* = 0.007) (>2 h OR: 1.91 95% CI: 1.09–3.36 *p* = 0.024) (Table 2).

Highers odds of stillbirth within the neonatal/birth domain included pre-term delivery (OR: 7.38 95% CI: 4.45–11.48 *p* < 0.001) compared with term delivery, membranes rupturing between >4 and ≤12 h before delivery (OR: 2.60 95% CI: 1.62–4.18 *p* < 0.001) compared with <1 h before delivery. Any duration of >12 h before delivery compared with <1 h highlighted a positive trend, yet no statistically significant association (Table 2).

### Associations With Stillbirth—Multivariable Analysis

The area type was associated with stillbirth independent of the following confounding variables: access to clean water, maternal education level, type of house, time traveled to the hospital, maternal disease, previous stillbirth, and household income (Supplementary Tables 7–13).

**Figure 4:**
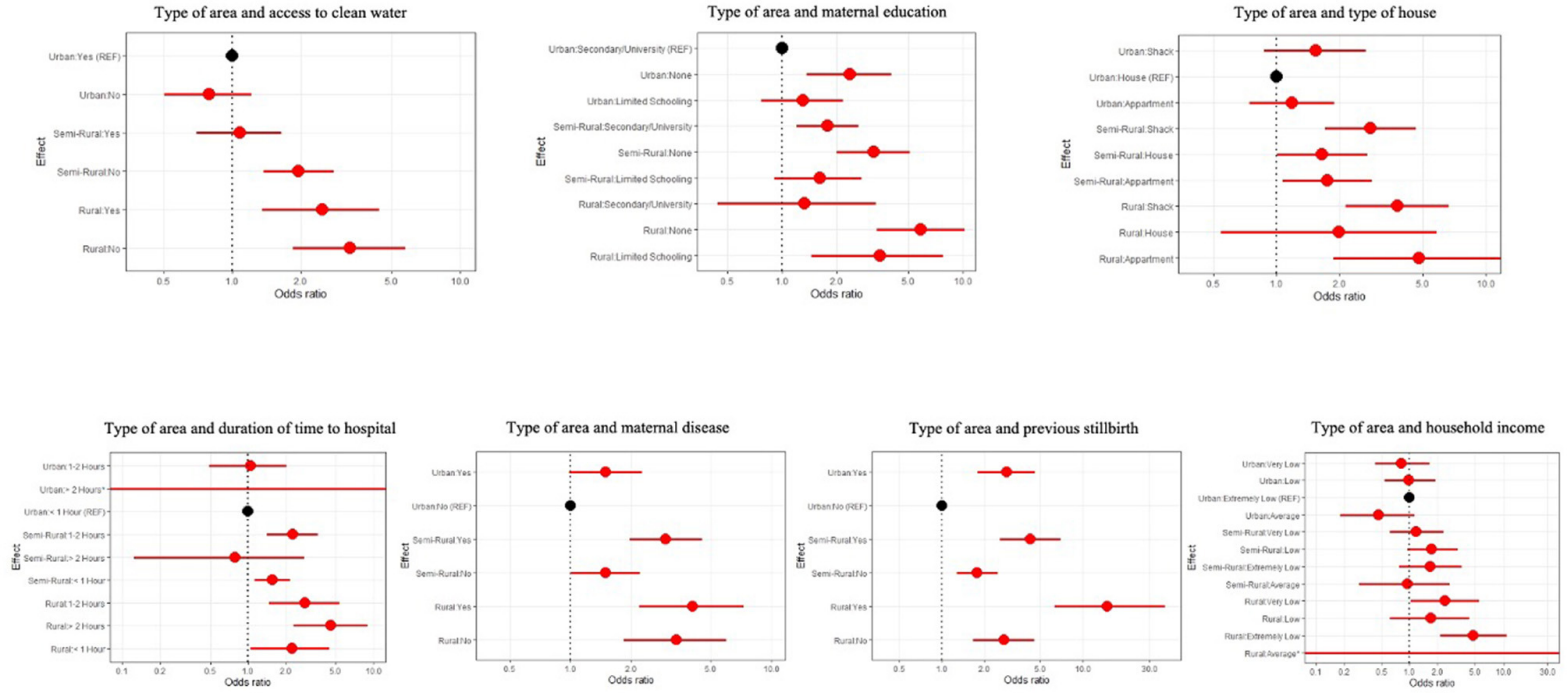
Forest plots for area type and the following confounding variables: access to clean water, maternal education, type of house, time traveled to the hospital, maternal disease, previous stillbirth(s), and household income.

Mothers living in a rural area with no access to clean water were over three times more likely to experience stillbirth than the mothers living in an urban area with access to clean water (OR: 3.3 95% CI: 1.87–5.82 *p* < 0.001), as shown in Figure 4. The interaction between education level and area type indicated that the mothers with no formal education had the highest odds of stillbirth, and this association was magnified for those in rural settings compared to mothers residing in an urban environment with secondary school education (OR: 5.86 95% CI: 3.33–10.24 *p* < 0.001) (Figure 4, Supplementary Table 8). The mothers living in a rural area living in a shack were almost four times more likely to have a stillbirth than the mothers from urban areas living in a house (OR: 3.78 9% CI: 2.15–6.64 *p* < 0.001) (Figure 4). The interaction analysis found that the mothers from rural settings with >2 h travel had the highest odds of stillbirth compared with the mothers living in an urban area with <1 h to travel (OR: 4.58 95% CI: 2.32–9.04 *p* < 0.001) (Figure 4). The mothers in a rural area with or without disease compared with the mothers who lived in an urban area without disease had higher odds of stillbirth (Figure 4), and those with a disease in a rural area had the highest odds of stillbirth (OR: 4.04 95% CI: 2.22–7.34 *p* < 0.001). The mothers with previous stillbirth(s) and living in a rural area were 15 times more likely to have a stillbirth compared with the mothers in an urban setting with no previous stillbirth (OR: 15.00% CI: 6.15–36.60 *p* < 0.001) (Figure 4). The mothers from an extremely low income household and living in a rural environment compared with those from an urban environment with extremely low income had the highest odds of stillbirth (OR: 4.75 95% CI: 2.13–10.92 *p* < 0.001) (Figure 4).

## Discussion

This article describes two studies, conducted over a combined period of five months including 539 stillbirths, in a large public tertiary hospital in Nigeria. An incidence of 180/1,000 births was identified in S2. Triangulation; the combination of two study designs, was used to strengthen the conclusions that can be drawn from any of the studies in isolation (19). The earliest report of stillbirths within Hausa communities was between 1974 and 1977 and reported an incidence of 113/1,000, suggesting that little has changed in 40 years (20). Reports from Uganda (120/1,000) and Ethiopia (71/1,000) indicate that other African regions also have an unacceptably high incidence of stillbirths (21, 22).

Overall, the following maternal factors were associated with higher odds of stillbirth: no formal maternal education, previous stillbirth(s), and having “any disease.” Hypertension was associated with higher odds of stillbirth, and it is also identified as a factor in perinatal mortality in Uganda and Cameroon (13, 21). Pre-term delivery and membranes rupturing >4 h prior to delivery were also associated with higher odds of stillbirth. A suitable intervention would be to educate/inform the mothers and families about the importance of getting to the hospital as quickly as possible after membranes rupture, to reduce the risk of maternal and neonatal infection.

The mothers from semi-rural and rural environments had higher odds of stillbirth than their urban counterparts. The association between rurality and stillbirth was independent of other key sociodemographic and living environment characteristics. The risks of some of these characteristics were amplified in rural settings, for example, rural mothers who had previously had a stillbirth(s) were found to be at high risk of delivering a subsequent stillbirth.

The Nigerian health system is split into federal and state and it is unclear where governmental support for pregnant women lies due to service differences (23). Furthermore, traditional practices, lack of knowledge, and/or a reluctance to engage in antenatal services have also been shown to affect maternal and neonatal health in rural Nigeria (23, 24). To further understand, it would be useful to compare the support and education provided to mothers in urban areas and what interventions have been successful as well as understanding whether it is an inequality in healthcare access or education, which renders these mothers at greater risk. A measure on community stillbirths is also essential to fully understand and obtain realistic rates. Therefore, assessment of community birth and stillbirth is warranted in future work.

Some conflicting findings were highlighted: maternal age; S1 identified that being <20 years was associated with higher odds of a stillbirth and S2 found that being aged ≥35 years was associated with higher odds of stillbirth. S1 found household income below average was associated with lower odds of stillbirth and S2 identified the opposite. S1 identified not having access to clean water had lower odds of stillbirth but in S2, not having access to clean water had higher odds. Conflicting findings cannot be explained without some speculation and consequently limit confirmation of logical assumptions. These findings may reflect study design (particularly selection and matching of controls in the S1) or chance findings, another option may be due to recall bias, where responses are based on subjective memory recall rather than objective records; accordingly, follow-up studies should involve home visits and/or photographic records that would mitigate any subjectivity. Equally, other counterintuitive findings, such as average income is associated with higher odds of stillbirth, also necessitate additional scrutiny to ratify the integrity of these data, although these could be attributed to a selection bias induced by the study design.

The key areas of focus for future work are to focus on hypertension throughout pregnancy, to review the existing interventions in similar demographic and geographic locations, and how hypertension is managed and monitored. In 2011, Jabeen reviewed the impact of calcium supplementation and aspirin use on maternal morbidity and eclampsia in high-risk cases, yet, despite maternal benefits, they determined that further work is needed to ascertain their benefits in relation to stillbirths (25). Improved approaches for maternal medical history data collection are needed; namely to review whether hypertension is pregnancy induced or pre-existing.

The rapid enrolment and quality of data collection were key strengths of the study, with no dropouts or withdrawals. Not only does this represent the excellent work of the research team, but it is also a demonstration of the understanding of the research need in this area by the mothers and healthcare workers in the hospital.

Multivariable analyses were conducted; however, the observational nature of the study design connotes that findings are not intended to be interpreted causally. Finally, while limiting our studies to a single site meant that the questions asked would have a relatively consistent interpretation, and findings cannot be generalised beyond this setting.

In conclusion, it is evident that stillbirth is a neglected public health concern, and the severity in Kano is highlighted when directly compared to the reported national average rate of 43/1,000 births (12); aggregated data often occlude the granularity and severity at the regional level. S2 identified an incidence four times worse than the reported rate; which is acknowledged to be the second highest stillbirth rate in the world (10). A similar rate was identified in a different hospital study in Northern Nigeria confirming this geographical location is an area of high need (20, 26). These initial studies identifying the magnitude of the problem have been successful in understanding the need for further and urgent work to be carried out.

We have made significant advances in understanding the magnitude of the problem being faced by expectant mothers and maternal healthcare workers in Kano. We identified a high incidence of stillbirth, which we believe still underestimates the true incidence as the studies were single-site and hospital-based. Given a large number of stillbirths, we were able to successfully investigate potential determinants and explore confounding relationships. This work, therefore, provides a platform for further work focussing on evidence-based intervention development and implementation.

## Data Availability Statement

Datasets specific to these studies will be made available upon request to the corresponding author.

## Ethics Statement

The studies involving human participants were reviewed and approved by Health Research Ethics Committee of the Ministry (of health), Kano, Nigeria. The participants provided informed consent to participate in this study.

## Author Contributions

RM wrote the manuscript, conducted overall study management, led the research from the UK perspective, collated and managed the data, and contributed to study design including questionnaire development. FZM contributed to the study design and led the research from a Nigerian perspective. WJW conducted the statistics on this study, contributed to study design, and contributed to writing the manuscript. DG contributed to writing the manuscript, oversight on the statistics, contributed to study design, and manuscript design. FIA, MB, FHS, AK, RYK, MYM, ASM, EO, and FMT contributed to sourcing data and data quality assurance. CPE contributed through training of the research assistants and contributing to data quality assurance. KH contributed via oversight of research integrity. KI contributed as local PI, responsible for the research in Nigeria. TRW was CI on the study and contributed to the writing of the manuscript. All authors contributed to the article and approved the submitted version.

## Funding

This collaboration was partially supported (S2) by funding from Health and Care Research Wales.

## Conflict of Interest

The authors declare that the research was conducted in the absence of any commercial or financial relationships that could be construed as a potential conflict of interest.

## Publisher's Note

All claims expressed in this article are solely those of the authors and do not necessarily represent those of their affiliated organizations, or those of the publisher, the editors and the reviewers. Any product that may be evaluated in this article, or claim that may be made by its manufacturer, is not guaranteed or endorsed by the publisher.

## Footnotes

^1^Milton R, Gillespie D, Dyer C, Taiyari K, Carvalho MJ, et al. Neonatal sepsis and mortality in low- and middle income countries from a facility-based birth cohort: an international multi-site prospective observational study. (unpublished).
