# Supplementary material for: Determinants of Stillbirth From Two Observational Studies Investigating Deliveries in Kano, Nigeria
Source: Front Glob Womens Health. 2022 Jan 14;2:788157. doi: 10.3389/fgwh.2021.788157 (PMC8795591; doi:10.3389/fgwh.2021.788157)
Supplement: Supplementary file 1 [file Data_Sheet_1.docx]

Appendix

**Supplementary Figure 1: Literature Search Results**

| Search criteria | Title / link | SB Rate Nigeria | Total number of Stillbirth | Total number of births | Setting | Notes |
| --- | --- | --- | --- | --- | --- | --- |
| Stillbirth and LMIC | [Understanding cause of stillbirth: a prospective observational multi-country study from sub-Saharan Africa.](https://eur03.safelinks.protection.outlook.com/?url=https%3A%2F%2Fpubmed.ncbi.nlm.nih.gov%2F31801488%2F&data=02%7C01%7Cmiltonrl1%40cardiff.ac.uk%7Cef09c2da42fd49c5710508d8330d04a9%7Cbdb74b3095684856bdbf06759778fcbc%7C1%7C0%7C637315478289657553&sdata=tZU8qsN%2B%2FzLDFEBE021eG48hUnnCYDTXzyhY1QHxKfQ%3D&reserved=0) | N/A |  |  | 12 hospitals in Kenya, Malawi, Sierra Leone and Zimbabwe. | This was a prospective, observational study where stillbirths (28 weeks or more) were reviewed to assign the cause of death by healthcare providers, an expert panel and by using computer-based algorithms. |
| Stillbirth and LMIC | [An Observational Pilot Study Evaluating the Utility of Minimally Invasive Tissue Sampling to Determine the Cause of Stillbirths in South African Women.](https://eur03.safelinks.protection.outlook.com/?url=https%3A%2F%2Fpubmed.ncbi.nlm.nih.gov%2F31598656%2F&data=02%7C01%7Cmiltonrl1%40cardiff.ac.uk%7Cef09c2da42fd49c5710508d8330d04a9%7Cbdb74b3095684856bdbf06759778fcbc%7C1%7C0%7C637315478289657553&sdata=qk7IiomcL%2BaIKex1nI%2BeHXNOGbLK2p8A%2Bf5JbrKl6AM%3D&reserved=0) | N/A |  |  | Chris Hani Baragwanath Academic Hospital (CHBAH), Soweto | Investigated the utility of minimally invasive tissue sampling (MITS), placental examination, and clinical history, in attributing the causes of stillbirth in a South African LMIC setting. |
| Stillbirth and LMIC | [Stillbirth rates in low-middle income countries 2010 - 2013: a population-based, multi-country study from the Global Network.](https://eur03.safelinks.protection.outlook.com/?url=https%3A%2F%2Fpubmed.ncbi.nlm.nih.gov%2F26063292%2F&data=02%7C01%7Cmiltonrl1%40cardiff.ac.uk%7Cef09c2da42fd49c5710508d8330d04a9%7Cbdb74b3095684856bdbf06759778fcbc%7C1%7C0%7C637315478289667544&sdata=AvM6jXrukQhRF%2FWUkpr4v9Y6A0ajAhVcEL0tptaG9kU%3D&reserved=0) | N/A |  |  | 7 sites in low-resource settings (Kenya, Zambia, India, Pakistan, Guatemala and Argentina). | A prospective, population-based observational study of all pregnant women in defined geographic areas across 7 sites in low-resource settings. |
| Stillbirth and LMIC | [Strategies to reduce and maintain low perinatal mortality in resource-poor settings - Findings from a four-decade observational study of birth records from a large public maternity hospital in Papua New Guinea.](https://eur03.safelinks.protection.outlook.com/?url=https%3A%2F%2Fpubmed.ncbi.nlm.nih.gov%2F30209806%2F&data=02%7C01%7Cmiltonrl1%40cardiff.ac.uk%7Cef09c2da42fd49c5710508d8330d04a9%7Cbdb74b3095684856bdbf06759778fcbc%7C1%7C0%7C637315478289667544&sdata=0ig1vUwU8mw8R%2Fz99Oa2DCDATmxQujaJ4nCNhBSgrPM%3D&reserved=0) | N/A |  |  | Port Moresby General Hospital in Papua New Guinea | Retrospective observational study to review birth mode and perinatal mortality in a large LMIC hospital with strict labour management protocols and expertise in vacuum extraction. |
| Stillbirth and LMIC | [Investigation of stillbirth causes in Suriname: application of the WHO ICD-PM tool to national-level hospital data.](https://www.tandfonline.com/doi/full/10.1080/16549716.2020.1794105) | N/A |  |  | All hospitals in Suriname | (1) assess characteristics and risk indicators of women with a stillbirth, (2) determine the timing and causes of stillbirths according to the ICD-PM with critical evaluation of its application and (3) propose recommendations for the reduction of stillbirths. |
| Stillbirth and Nigeria | [Prevalence and determinants of stillbirth in Nigerian referral hospitals: a multicentre study.](https://eur03.safelinks.protection.outlook.com/?url=https%3A%2F%2Fpubmed.ncbi.nlm.nih.gov%2F31888536%2F&data=02%7C01%7Cmiltonrl1%40cardiff.ac.uk%7Cfa44e37e1c5342d4010208d8330f3f2a%7Cbdb74b3095684856bdbf06759778fcbc%7C1%7C0%7C637315487872924681&sdata=%2FuSL8TSAB4DBq834ZEqvfHZ9gb8SoYfPvDgA6byb9vs%3D&reserved=0) | 39·6/1000 | 175 | 4416 | Six general hospitals (4 south and 2 north), and two teaching hospitals (both north) in Nigeria. | A cross-sectional study of all deliveries over a period of 6 months |
| Stillbirth and Nigeria | [Prevalence and maternal socio-demographic factors associated with stillbirth in health facilities in Anambra, South-East Nigeria.](https://eur03.safelinks.protection.outlook.com/?url=https%3A%2F%2Fpubmed.ncbi.nlm.nih.gov%2F32127881%2F&data=02%7C01%7Cmiltonrl1%40cardiff.ac.uk%7Cfa44e37e1c5342d4010208d8330f3f2a%7Cbdb74b3095684856bdbf06759778fcbc%7C1%7C0%7C637315487872924681&sdata=IdC1%2FVj2Q4WBnUtyAB2PSOeZAp8nfn%2B12%2Bv%2FRnrgElE%3D&reserved=0) | 19·76/1000 | 313 | 15844 | Rural communities | To determine the prevalence of stillbirth and also explore the maternal socio-demographic factors associated with stillbirth among mothers in rural communities in Anambra Central Senatorial District of Anambra State Nigeria who gave birth between January 2012 and December 2016. |
| Stillbirth and Nigeria | [Tracking stillbirths by referral pattern and causes in a rural tertiary hospital in Southern Nigeria.](https://eur03.safelinks.protection.outlook.com/?url=https%3A%2F%2Fpubmed.ncbi.nlm.nih.gov%2F30027919%2F&data=02%7C01%7Cmiltonrl1%40cardiff.ac.uk%7Cfa44e37e1c5342d4010208d8330f3f2a%7Cbdb74b3095684856bdbf06759778fcbc%7C1%7C0%7C637315487872934673&sdata=7UxUgORYxG9S5oGAZXC1xf0tPDVs101XLJc2ZLACXKg%3D&reserved=0) | 175/1000 | 218 | 1243 | Madonna University Teaching Hospital, Rivers State, Nigeria | This study evaluated the causes and determinants of stillbirth by the referral pattern in a rural area in southern Nigeria. This was a retrospective case–controlled study of stillbirth |
| Stillbirth and Nigeria | [Stillbirth rate at an emerging tertiary health institution in Enugu, southeast Nigeria.](https://eur03.safelinks.protection.outlook.com/?url=https%3A%2F%2Fpubmed.ncbi.nlm.nih.gov%2F21872234%2F&data=02%7C01%7Cmiltonrl1%40cardiff.ac.uk%7Cfa44e37e1c5342d4010208d8330f3f2a%7Cbdb74b3095684856bdbf06759778fcbc%7C1%7C0%7C637315487872934673&sdata=FozHquZKYN3gSGTtNqkiz8V2cTRIrtTGXVJcj3eqZdI%3D&reserved=0) | 74/1000 | 153 | 2064 | Enugu State University of Science and Technology Teaching Hospital, Parklane, Nigeria | To determine the stillbirth rate and factors predisposing to a stillbirth delivery, aim of identifying solutions. The sociodemographic characteristics of the mothers were documented and the possible causes of death were analyzed. |
| Stillbirth and Nigeria | [An assessment of stillbirths in a tertiary hospital in northern Nigeria.](https://eur03.safelinks.protection.outlook.com/?url=https%3A%2F%2Fpubmed.ncbi.nlm.nih.gov%2F25204335%2F&data=02%7C01%7Cmiltonrl1%40cardiff.ac.uk%7Cfa44e37e1c5342d4010208d8330f3f2a%7Cbdb74b3095684856bdbf06759778fcbc%7C1%7C0%7C637315487872954661&sdata=OMlbZ3zT%2FOB8ak3GWl29MGISZn9GW4mr7cJVvLno4X8%3D&reserved=0) | 170/1000 | 761 | 4479 | Birnin Kudu, North-west, Nigeria. | determine the stillbirth rate and causes in Birnin Kudu, North-west, Nigeria. |
| Stillbirth and Nigeria | [Determinants of perinatal mortality in Nigeria.](https://eur03.safelinks.protection.outlook.com/?url=https%3A%2F%2Fpubmed.ncbi.nlm.nih.gov%2F21489535%2F&data=02%7C01%7Cmiltonrl1%40cardiff.ac.uk%7Cfa44e37e1c5342d4010208d8330f3f2a%7Cbdb74b3095684856bdbf06759778fcbc%7C1%7C0%7C637315487872974652&sdata=uqePWaI1vKUtx1hd6f%2BAJGb7EoJh3GazQolMLfnzhYI%3D&reserved=0) | 71/1000 | 651 | 9208 | the Federal Capital Territory (FCT), Katsina and Lagos states were selected for the study. 21 health facilities (7 from each state) | To determine risk factors for perinatal mortality among hospital-based deliveries in Nigeria. |
| Stillbirth and Nigeria | [Stillbirth rate in a teaching hospital in South-eastern Nigeria: a silent tragedy.](https://eur03.safelinks.protection.outlook.com/?url=https%3A%2F%2Fpubmed.ncbi.nlm.nih.gov%2F23439739%2F&data=02%7C01%7Cmiltonrl1%40cardiff.ac.uk%7Cfa44e37e1c5342d4010208d8330f3f2a%7Cbdb74b3095684856bdbf06759778fcbc%7C1%7C0%7C637315487872984646&sdata=orhVJaSzPaGOjub6T7yDmn34zyOV%2F%2Bl8sLfoK3lkepY%3D&reserved=0) | 180/1000 | 206 | 1142 | Teaching Hospital in South-Eastern Nigeria | The objective of this study was to determine the stillbirth rate at the Imo State University Teaching Hospital, Orlu. |
| Stillbirth and Nigeria | [Determinants of stillbirths in katsina, Nigeria: a hospital-based study.](https://eur03.safelinks.protection.outlook.com/?url=https%3A%2F%2Fpubmed.ncbi.nlm.nih.gov%2F25918622%2F&data=02%7C01%7Cmiltonrl1%40cardiff.ac.uk%7Cfa44e37e1c5342d4010208d8330f3f2a%7Cbdb74b3095684856bdbf06759778fcbc%7C1%7C0%7C637315487872994638&sdata=b3v0A%2FWhfUUXuA2dCdXJm4prr3fK%2BCyZWPFPOshq0N4%3D&reserved=0) | 49·9/1000 | 331 | 6628 | Federal Medical Centre Katsina, located in north western Nigeria. | The aim was to determine the stillbirth rate and its determinants. This study was a retrospective review of all deliveries conducted between 1st March 2010 and 31st December 2012. |
| Stillbirth and Nigeria | [Perinatal Mortality in a Northwestern Nigerian City: A Wake up Call.](https://eur03.safelinks.protection.outlook.com/?url=https%3A%2F%2Fpubmed.ncbi.nlm.nih.gov%2F25340044%2F&data=02%7C01%7Cmiltonrl1%40cardiff.ac.uk%7Cfa44e37e1c5342d4010208d8330f3f2a%7Cbdb74b3095684856bdbf06759778fcbc%7C1%7C0%7C637315487872994638&sdata=WGkKtsZ6bEbc%2FfQ%2BWdtr19W4Bk%2BtAt%2BhXYWOi4yOsyQ%3D&reserved=0) | 85/1000 | 94 | 1104 | Three major public hospitals in Katsina | Data were collected over a 6week period on maternal socio-demographic, antenatal, and delivery variables. Data were similarly obtained on neonatal profile and morbidities. |
| Stillbirth and Nigeria | [Applying the WHO ICD-PM classification system to stillbirths in a major referral Centre in Northeast Nigeria: A retrospective analysis from 2010-2018.](http://ovidsp.dc1.ovid.com.abc.cardiff.ac.uk/ovid-b/ovidweb.cgi?&S=GELLFPDHMEACCMMEKPAKMEDKHKPDAA00&Complete+Reference=S.sh.44%7c6%7c1&Counter5=SS_view_found_complete%7c632195453%7cemczd%7cembase%7cemexb&Counter5Data=632195453%7cemczd%7cembase%7cemexb) | 55/1000 | 1177 | 21462 | Major referal centre in north-east nigeria. | This was a retrospective observational study in a major referral centre in northeast Nigeria between 2010 and 2018. Specialists assigned causes of stillbirths after an extensive audit of available stillbirths’ records. Cause of death was assigned via consensus using the ICD-PM classification system. |
| Stillbirth and Determinant and LMIC | [Trend of stillbirth rates and the associated risk factors in babol, northern iran.](https://eur03.safelinks.protection.outlook.com/?url=https%3A%2F%2Fpubmed.ncbi.nlm.nih.gov%2F24498477%2F&data=02%7C01%7Cmiltonrl1%40cardiff.ac.uk%7C95d6cdedcf1f40c95b0008d8447d7126%7Cbdb74b3095684856bdbf06759778fcbc%7C1%7C0%7C637334652857607382&sdata=LHd5vgywLP9ltp5jZJK3%2B%2FgyPreBj3KYNbxQBy1%2BZZo%3D&reserved=0) | N/A |  |  | two major Gynecological wards in Shahid Yahyanejat and Babol clinic hospitals in Babol, Northern Iran | A retrospective study was conducted based on the data of hospital charts |

**Supplementary Table 1:** *Study 1 - Area type and access to clean water (Case-control)*

| Stillbirth modelled on area and water – main terms only | | | | | | | |
| --- | --- | --- | --- | --- | --- | --- | --- |
|  |  | B | SE | P Value | OR | CI - OR | |
|  |  |  |  |  |  | Low | High |
| Type of area | Rural | 2·416 | 0·474 | <0·001 | 11·200 | 4·423 | 28·359 |
|  | Semi-Rural | 1·348 | 0·267 | <0·001 | 3·849 | 2·281 | 6·497 |
|  | Urban | Reference category | | | | | |
| Access to clean water | No | -2·516 | 0·322 | <0·001 | 0·081 | 0·043 | 0·152 |
|  | Yes | Reference category | | | | | |
| Stillbirth modelled on area and water – interactions only | | | | | | | |
|  |  | B | SE | P Value | OR | CI - OR | |
|  |  |  |  |  |  | Low | High |
| Type of area *  Access to clean water | Rural*No | -0·0062 | 0·5134 | 0·990 | 0·9938 | 0·3633 | 2·7186 |
|  | Rural*Yes^+^ | 18·579 | 5752·4720 | 0·997 | 117177249·5590 | 0·0000 | Inf |
|  | Semi-Rural*No | -1·1034 | 0·3425 | 0·001 | 0·3318 | 0·1695 | 0·6492 |
|  | Semi-Rural*Yes | 2·0518 | 0·7658 | 0·007 | 7·7820 | 1·7349 | 34·9063 |
|  | Urban*No | -2·3171 | 0·3518 | <0·001 | 0·0986 | 0·0495 | 0·1964 |
|  | Urban*Yes | Reference category | | | | | |

^+^*coefficients and confidence intervals are not estimatable due to insufficient responses*

**Supplementary Table 2:** *Study 1 - Area type and maternal education (Case-control)*

| Stillbirth modelled on area and mother’s education – main terms only | | | | | | | | |
| --- | --- | --- | --- | --- | --- | --- | --- | --- |
|  |  | B | SE | | P Value | OR | CI - OR | |
|  |  |  |  | |  |  | Low | High |
| Type of area | Rural | 1·590 | 0·409 | | <0·001 | 4·903 | 2·199 | 10·933 |
|  | Semi-Rural | 0·734 | 0·220 | | 0·001 | 2·084 | 1·354 | 3·207 |
|  | Urban | Reference category | | | | | | |
| Mother’s education level | None | 0·884 | 0·276 | | 0·001 | 2·421 | 1·409 | 4·160 |
|  | Limited | 0·029 | 0·229 | | 0·9 | 1·029 | 0·657 | 1·614 |
|  | Secondary/University | Reference category | | | | | | |
| Stillbirth modelled on area and mother’s education – interactions only | | | | | | | | |
|  |  | B | | SE | P Value | OR | CI - OR | |
|  |  |  | |  |  |  | Low | High |
| Type of Area * Mother’s education level | Rural*None | 2·323 | | 0·525 | <0·001 | 10·202 | 3·645 | 28·549 |
|  | Rural*Limited | 1·642 | | 0·642 | 0·011 | 5·164 | 1·467 | 18·171 |
|  | Rural*Secondary/University | 2·189 | | 0·900 | 0·015 | 8·924 | 1·529 | 52·086 |
|  | Semi-Rural*None | 1·752 | | 0·376 | <0·001 | 5·766 | 2·762 | 12·038 |
|  | Semi-Rural*Limited | 0·715 | | 0·298 | 0·016 | 2·045 | 1·140 | 3·666 |
|  | Semi-Rural*Secondary/University | 0·757 | | 0·289 | 0·009 | 2·131 | 1·210 | 3·755 |
|  | Urban*None | 0·894 | | 0·408 | 0·029 | 2·444 | 1·098 | 5·440 |
|  | Urban*Limited | 0·134 | | 0·332 | 0·687 | 1·143 | 0·596 | 2·193 |
|  | Urban*Secondary/University | Reference category | | | | | | |

**Supplementary Table 3:** *Study 1 - Area type and type of house (Case-control)*

| SB modelled on area and house type – main terms only | | | | | | | | |
| --- | --- | --- | --- | --- | --- | --- | --- | --- |
|  |  | B | SE | | P Value | OR | CI - OR | |
|  |  |  |  | |  |  | Low | High |
| Type of area | Rural | 1·878 | 0·392 | | <0·001 | 6·539 | 3·031 | 14·105 |
|  | Semi-Rural | 0·865 | 0·205 | | <0·001 | 2·375 | 1·588 | 3·552 |
|  | Urban | Reference category | | | | | | |
| House type | Shack | 0·084 | 0·246 | | 0·734 | 1·087 | 0·671 | 1·761 |
|  | Apartment | 0·128 | 0·225 | | 0·569 | 1·137 | 0·731 | 1·766 |
|  | House | Reference category | | | | | | |
| Stillbirth modelled on area and house type – interactions only | | | | | | | | |
|  |  | B | | SE | P Value | OR | CI – OR | |
|  |  |  | |  |  |  | Low | High |
| Type of area * House type | Rural*Shack | 2·179 | | 0·528 | <0·001 | 8·841 | 3·143 | 24·865 |
|  | Rural*Apartment^+^ | 19·648 | | 5095 | 0·997 | - | 0·000 | Inf |
|  | Rural*House | 0·096 | | 0·710 | 0·892 | 1·101 | 0·274 | 4·424 |
|  | Semi-Rural*Shack | 0·848 | | 0·358 | 0·018 | 2·336 | 1·159 | 4·708 |
|  | Semi-Rural*Apartment | 0·918 | | 0·329 | 0·005 | 2·503 | 1·314 | 4·767 |
|  | Semi-Rural*House | 0·705 | | 0·323 | 0·029 | 2·024 | 1·074 | 3·815 |
|  | Urban*Shack | -0·191 | | 0·359 | 0·594 | 0·826 | 0·409 | 1·668 |
|  | Urban*Apartment | -0·099 | | 0·282 | 0·725 | 0·906 | 0·521 | 1·574 |
|  | Urban*House | Reference category | | | | | | |

^+^*coefficients and confidence intervals are not estimatable due to insufficient responses*

**Supplementary Table 4:** *Study 1 - Area type and maternal disease (Case-control)*

| Stillbirth modelled on area and maternal disease – main terms only | | | | | | | |
| --- | --- | --- | --- | --- | --- | --- | --- |
|  |  | B | SE | P Value | OR | CI – OR | |
|  |  |  |  |  |  | Low | High |
| Type of area | Rural | 2·103 | 0·405 | <0·001 | 8·195 | 3·703 | 18·135 |
|  | Semi-Rural | 0·863 | 0·221 | <0·001 | 2·370 | 1·537 | 3·656 |
|  | Urban | Reference category | | | | | |
| Maternal disease | Yes | 1·317 | 0·229 | <0·001 | 3·734 | 2·382 | 5·852 |
|  | No | Reference category | | | | | |
| Stillbirth modelled on area and maternal disease – interactions only | | | | | | | |
|  |  | B | SE | P Value | OR | CI - OR | |
|  |  |  |  |  |  | Low | High |
| Type of Area *  Maternal disease | Rural*Yes | 3·209 | 0·679 | <0·001 | 24·757 | 6·539 | 93·730 |
|  | Rural*No | 1·911 | 0·501 | <0·001 | 6·761 | 2·534 | 18·039 |
|  | Semi-Rural*Yes | 2·175 | 0·339 | <0·001 | 8·803 | 4·526 | 17·121 |
|  | Semi-Rural*No | 0·389 | 0·359 | 0·279 | 1·475 | 0·730 | 2·983 |
|  | Urban*Yes | 1·020 | 0·295 | 0·001 | 2·774 | 1·557 | 4·944 |
|  | Urban*No | Reference category | | | | | |

**Supplementary Table 5:** *Study 1 - Area type and previous stillbirth(s) (Case-control)*

| Stillbirth modelled on area and previous stillbirth(s) – main terms only | | | | | | | |
| --- | --- | --- | --- | --- | --- | --- | --- |
|  |  | B | SE | P Value | OR | CI - OR | |
|  |  |  |  |  |  | Low | High |
| Type of area | Rural | 1·896 | 0·389 | <0·001 | 6·662 | 3·107 | 14·283 |
|  | Semi-Rural | 0·807 | 0·211 | <0·001 | 2·241 | 1·483 | 3·386 |
|  | Urban | Reference category | | | | | |
| Has the mother had a previous stillbirth? | Yes | 0·939 | 0·231 | <0·001 | 2·557 | 1·627 | 4·020 |
|  | No | Reference category | | | | | |
| Stillbirth modelled on area and previous stillbirth(s) – interactions only | | | | | | | |
|  |  | B | SE | P Value | OR | CI - OR | |
|  |  |  |  |  |  | Low | High |
| Type of area * Has the mother had a previous stillbirth? | Rural*Yes | 1·686 | 0·582 | 0·004 | 5·397 | 1·725 | 16·887 |
|  | Rural*No | 2·564 | 0·530 | <0·001 | 12·985 | 4·593 | 36·707 |
|  | Semi-Rural*Yes | 1·844 | 0·372 | <0·001 | 6·324 | 3·048 | 13·120 |
|  | Semi-Rural*No | 0·785 | 0·234 | 0·001 | 2·192 | 1·385 | 3·471 |
|  | Urban*Yes | 1·215 | 0·350 | 0·001 | 3·370 | 1·698 | 6·690 |
|  | Urban*No | Reference category | | | | | |

**Supplementary Table 6:** *Study 1 - Area type and household income (Case-control)*

| Stillbirth modelled on area and income band – main terms only | | | | | | | |
| --- | --- | --- | --- | --- | --- | --- | --- |
|  |  | B | SE | P Value | OR | CI - OR | |
|  |  |  |  |  |  | Low | High |
| Type of area | Rural | 2·355 | 0·409 | <0·001 | 10·540 | 4·727 | 23·503 |
|  | Semi-Rural | 1·234 | 0·240 | <0·001 | 3·435 | 2·146 | 5·496 |
|  | Urban | Reference category | | | | | |
| Income band | Average | 2·688 | 0·546 | <0·001 | 14·695 | 5·044 | 42·817 |
|  | Low | 1·295 | 0·274 | <0·001 | 3·651 | 2·136 | 6·241 |
|  | Very Low | 0·567 | 0·277 | 0·040 | 1·763 | 1·025 | 3·034 |
|  | Extremely Low | Reference category | | | | | |
| Stillbirth modelled on area and income band – interactions only | | | | | | | |
|  |  | B | SE | P Value | OR | CI - OR | |
|  |  |  |  |  |  | Low | High |
| Type of area * Income band | Rural*Average^+^ | 23·249 | 40192·970 | 0·999 | 12501312353.000 | 0.000 | Inf |
|  | Rural*Low^+^ | 22·623 | 7130·288 | 0·997 | 6685734405.000 | 0.000 | Inf |
|  | Rural*Very Low | 3·700 | 1·233 | 0·003 | 40·446 | 3·606 | 453·665 |
|  | Rural*Extremely Low | 3·131 | 0·563 | <0·001 | 22·901 | 7·599 | 69·020 |
|  | Semi-Rural*Average^+^ | 40·950 | 11912·69 | 0·997 | - | 0 | Inf |
|  | Semi-Rural*Low | 2·530 | 0·571 | <0·001 | 12·559 | 4·0979 | 38·488 |
|  | Semi-Rural*Very Low | 1·929 | 0·512 | <0·001 | 6·882 | 2·525 | 18·757 |
|  | Semi-Rural*Extremely Low | 2·517 | 0·434 | <0·001 | 12·388 | 5·296 | 28·974 |
|  | Urban*Average | 3·438 | 0·646 | <0·001 | 31·111 | 8·769 | 110·380 |
|  | Urban*Low | 2·395 | 0·452 | <0·001 | 10·973 | 4·524 | 26·616 |
|  | Urban*Very Low | 2·046 | 0·482 | <0·001 | 7·739 | 3·007 | 19·914 |
|  | Urban*Extremely Low | Reference category | | | | | |

^+^*coefficients and confidence intervals are not estimatable due to insufficient responses*

**Supplementary Table 7:** *Study 2 - Area type and access to clean water (Cohort)*

| Stillbirth modelled on area and water – main terms only | | | | | | | |
| --- | --- | --- | --- | --- | --- | --- | --- |
|  |  | B | SE | P Value | OR | CI - OR | |
|  |  |  |  |  |  | Low | High |
| Type of area | Rural | 1·108 | 0·216 | <0·001 | 3·028 | 1·982 | 4·625 |
|  | Semi-Rural | 0·480 | 0·150 | 0·001 | 1·616 | 1·203 | 2·169 |
|  | Urban | Reference category | | | | | |
| Access to clean water | No | 0·200 | 0·140 | 0·154 | 1·221 | 0·928 | 1·607 |
|  | Yes | Reference category | | | | | |
| Stillbirth modelled on area and water – interactions only | | | | | | | |
|  |  | B | SE | P Value | OR | CI - OR | |
|  |  |  |  |  |  | Low | High |
| Type of area *  Access to clean water | Rural*No | 1·193 | 0·290 | <0·001 | 3·297 | 1·869 | 5·816 |
|  | Rural*Yes | 0·909 | 0·302 | 0·003 | 2·481 | 1·374 | 4·480 |
|  | Semi-Rural*No | 0·672 | 0·181 | <0·001 | 1·959 | 1·374 | 2·792 |
|  | Semi-Rural*Yes | 0·074 | 0·219 | 0·737 | 1·076 | 0·701 | 1·654 |
|  | Urban*No | -0·238 | 0·225 | 0·289 | 0·788 | 0·508 | 1·224 |
|  | Urban*Yes | Reference category | | | | | |

**Supplementary Table 8:** *Study 2 - Area type and maternal education (Cohort)*

| Stillbirth modelled on area and mother’s education – main terms only | | | | | | | |
| --- | --- | --- | --- | --- | --- | --- | --- |
|  |  | B | SE | P Value | OR | CI - OR | |
| Type of Area | Rural | 0·833 | 0·227 | <0·001 | 2·299 | 1·474 | 3·588 |
|  | Semi-Rural | 0·428 | 0·150 | 0·004 | 1·535 | 1·144 | 2·059 |
|  | Urban | Reference category | | | | | |
| Mother’s education level | None | 0·787 | 0·168 | <0·001 | 2·200 | 1·580 | 3·050 |
|  | Limited | 0·162 | 0·183 | 0·377 | 1·18 | 0·816 | 1·670 |
|  | Secondary/University | Reference category | | | | | |
| Stillbirth modelled on area and mother’s education – interactions only | | | | | | | |
|  |  | B | SE | P Value | OR | CI - OR | |
|  |  |  |  |  |  | Low | High |
| Type of area * Mother’s education level | Rural*None | 1·767 | 0·286 | <0·001 | 5·852 | 3·330 | 10·239 |
|  | Semi-Rural*None | 1·166 | 0·235 | <0·001 | 3·209 | 2·017 | 5·072 |
|  | Urban*None | 0·864 | 0·275 | 0·002 | 2·372 | 1·363 | 4·024 |
|  | Rural*Limited Schooling | 1·247 | 0·424 | 0·003 | 3·480 | 1·449 | 7·793 |
|  | Semi-Rural*Limited Schooling | 0·477 | 0·281 | 0·090 | 1·611 | 0·910 | 2·757 |
|  | Urban*Limited Schooling | 0·268 | 0·264 | 0·309 | 1·308 | 0·767 | 2·167 |
|  | Rural*Secondary/University | 0·288 | 0·503 | 0·568 | 1·333 | 0·441 | 3·302 |
|  | Semi-Rural*Secondary/University | 0·577 | 0·200 | 0·004 | 1·780 | 1·201 | 2·636 |
|  | Urban*Secondary/University | Reference category | | | | | |

**Supplementary Table 9:** *Study 2 - Area type and type of house (Cohort)*

| Stillbirth modelled on area and house type – main terms only | | | | | | | | |
| --- | --- | --- | --- | --- | --- | --- | --- | --- |
|  |  | B | SE | | P Value | OR | CI - OR | |
|  |  |  |  | |  |  | Low | High |
| Type of area | Rural | 0·945 | 0·229 | | <0·001 | 2·573 | 1·644 | 4·027 |
|  | Semi-Rural | 0·489 | 0·148 | | 0·001 | 1·631 | 1·219 | 2·181 |
|  | Urban | Reference category | | | | | | |
| House type | Shack | 0·473 | 0·180 | | 0·009 | 1·604 | 1·128 | 2·282 |
|  | Apartment | 0·163 | 0·168 | | 0·333 | 1·177 | 0·846 | 1·636 |
|  | House | Reference category | | | | | | |
| Stillbirth modelled on area and house type – interactions only | | | | | | | | |
|  |  | B | | SE | P Value | OR | CI - OR | |
|  |  |  | |  |  |  | Low | High |
| Type of area * House type | Rural*Shack | 1·330 | | 0·288 | <0·001 | 3·781 | 2·152 | 6·644 |
|  | Rural*Apartment | 1·564 | | 0·462 | 0·001 | 4·778 | 1·933 | 11·811 |
|  | Rural*House | 0·684 | | 0·589 | 0·246 | 1·982 | 0·624 | 6·291 |
|  | Semi-Rural*Shack | 1·028 | | 0·253 | <0·001 | 2·796 | 1·701 | 4·594 |
|  | Semi-Rural*Apartment | 0·557 | | 0·250 | 0·026 | 1·746 | 1·070 | 2·848 |
|  | Semi-Rural*House | 0·499 | | 0·252 | 0·048 | 1·647 | 1·004 | 2·700 |
|  | Urban*Shack | 0·430 | | 0·285 | 0·13 | 1·538 | 0·880 | 2·686 |
|  | Urban*Apartment | 0·164 | | 0·238 | 0·491 | 1·178 | 0·739 | 1·876 |
|  | Urban*House | Reference category | | | | | | |

**Supplementary Table 10:** *Study 2 - Area type and time travelled to hospital (Cohort)*

| Stillbirth modelled on area and time travelled to hospital – main terms only | | | | | | | |
| --- | --- | --- | --- | --- | --- | --- | --- |
|  |  | B | SE | P Value | OR | CI - OR | |
|  |  |  |  |  |  | Low | High |
| Type of area | Rural | 1·036 | 0·245 | <0·001 | 2·818 | 1·745 | 4·550 |
|  | Semi-Rural | 0·494 | 0·149 | 0·001 | 1·639 | 1·223 | 2·197 |
|  | Urban | Reference category | | | | | |
| Time to hospital | >2 hours | 0·127 | 0·323 | 0·693 | 1·136 | 0·603 | 2·137 |
|  | 1-2 hours | 0·22 | 0·184 | 0·233 | 1·245 | 0·868 | 1·787 |
|  | <1 hour | Reference category | | | | | |
| Stillbirth modelled on area and time to hospital – interactions only | | | | | | | |
|  |  | B | SE | P Value | OR | CI - OR | |
|  |  |  |  |  |  | Low | High |
| Type of area * Time to hospital | Rural*>2 hours | 1·522 | 0·346 | <0·001 | 4·583 | 2·324 | 9·038 |
|  | Rural*1-2 hours | 1·052 | 0·330 | 0·001 | 2·865 | 1·500 | 5·472 |
|  | Rural*<1 hour | 0·805 | 0·368 | 0·029 | 2·236 | 1·088 | 4·598 |
|  | Semi-Rural*>2 hours | -0·239 | 0·758 | 0·753 | 0·788 | 0·178 | 3·480 |
|  | Semi-Rural*1-2 hours | 0·823 | 0·240 | 0·001 | 2·278 | 1·424 | 3·644 |
|  | Semi-Rural*< 1 hour | 0·446 | 0·164 | 0·006 | 1·562 | 1·133 | 2·152 |
|  | Urban*>2 hours^+^ | -19·992 | 17419·410 | 0·999 |  |  |  |
|  | Urban*1-2 hours | 0·049 | 0·359 | 0·891 | 1·050 | 0·520 | 2·122 |
|  | Urban*<1 hour | Reference category | | | | | |

^+^*coefficients and confidence intervals are not estimatable due to insufficient responses*

**Supplementary Table 11:** *Study 2 - Area type and maternal disease (Cohort)*

| Stillbirth modelled on area and maternal disease – main terms only | | | | | | | |
| --- | --- | --- | --- | --- | --- | --- | --- |
|  |  | B | SE | P Value | OR | CI - OR | |
|  |  |  |  |  |  | Low | High |
| Type of area | Rural | 1·096 | 0·217 | <0·001 | 2·991 | 1·956 | 4·574 |
|  | Semi-Rural | 0·529 | 0·148 | <0·001 | 1·697 | 1·269 | 2·268 |
|  | Urban | Reference category | | | | | |
| Maternal disease | Yes | 0·499 | 0·139 | <0·001 | 1·647 | 1·255 | 2·162 |
|  | No | Reference category | | | | | |
| Stillbirth modelled on area and maternal disease– interactions only | | | | | | | |
|  |  | B | SE | P Value | OR | CI - OR | |
|  |  |  |  |  |  | Low | High |
| Type of area *  Maternal disease | Rural*Yes | 1·396 | 0·305 | <0·001 | 4·038 | 2·223 | 7·337 |
|  | Rural*No | 1·209 | 0·297 | <0·001 | 3·351 | 1·871 | 6·002 |
|  | Semi-Rural*Yes | 1·092 | 0·209 | <0·001 | 2·979 | 1·979 | 4·487 |
|  | Semi-Rural*No | 0·402 | 0·198 | 0·044 | 1·495 | 1·011 | 2·211 |
|  | Urban*Yes | 0·405 | 0·211 | 0·055 | 1·500 | 0·992 | 2·268 |
|  | Urban*No | Reference category | | | | | |

**Supplementary Table 12:** *Study 2 - Area type and previous stillbirth(s) (Cohort)*

| Stillbirth modelled on area and previous stillbirth – main terms only | | | | | | | |
| --- | --- | --- | --- | --- | --- | --- | --- |
|  |  | B | SE | P Value | OR | CI - OR | |
|  |  |  |  |  |  | Low | High |
| Type of area | Rural | 1·149 | 0·2197 | <0·001 | 3·157 | 2·052 | 4·855 |
|  | Semi-Rural | 0·531 | 0·1498 | <0·001 | 1·701 | 1·268 | 2·281 |
|  | Urban | Reference category | | | | | |
| Has the mother had a previous stillbirth? | Yes | 1·055 | 0·1611 | <0·001 | 2·872 | 2·095 | 3·939 |
|  | No | Reference category | | | | | |
| Stillbirth modelled on area and previous stillbirth – interactions only | | | | | | | |
|  |  | B | SE | P Value | OR | CI - OR | |
|  |  |  |  |  |  | Low | High |
| Type of area * Has the mother had a previous stillbirth? | Rural*Yes | 2·708 | 0·455 | <0·001 | 15.000 | 6·148 | 36·596 |
|  | Rural*No | 1·019 | 0·256 | <0·001 | 2·769 | 1·678 | 4·569 |
|  | Semi-Rural*Yes | 1·451 | 0·252 | <0·001 | 4·267 | 2·605 | 6·987 |
|  | Semi-Rural*No | 0·577 | 0·173 | 0·001 | 1·780 | 1·269 | 2·497 |
|  | Urban*Yes | 1·065 | 0·238 | <0·001 | 2·901 | 1·819 | 4·627 |
|  | Urban*No | Reference category | | | | | |

**Supplementary Table 13:** *Study 2 - Area type and household income (Cohort)*

| Stillbirth modelled on area and income – main terms only | | | | | | | |
| --- | --- | --- | --- | --- | --- | --- | --- |
|  |  | B | SE | P Value | OR | CI - OR | |
| Type of area | Rural | 1·054 | 0·2205 | <0·001 | 2·868 | 1·862 | 4·418 |
|  | Semi-Rural | 0·489 | 0·1484 | 0·001 | 1·63 | 1·219 | 2·181 |
|  | Urban | Reference Category | | | | | |
| Income band | Average | -0·923 | 0·3369 | 0·006 | 0·397 | 0·205 | 0·769 |
|  | Low | -0·173 | 0·1949 | 0·374 | 0·841 | 0·574 | 1·232 |
|  | Very Low | -0·405 | 0·2027 | 0·046 | 0·667 | 0·448 | 0·992 |
|  | Extremely Low | Reference Category | | | | | |
| Stillbirth modelled on area and income – interactions only | | | | | | | |
|  |  | B | SE | P Value | OR | CI - OR | |
|  |  |  |  |  |  | Low | High |
| Type of area * Income band | Rural*Average^+^ | -12·867 | 394·775 | 0·974 | 0·000 | 0·000 | - |
|  | Semi-Rural*Average | -0·059 | 0·560 | 0·916 | 0·943 | 0·286 | 2·680 |
|  | Urban*Average | -0·765 | 0·463 | 0·099 | 0·465 | 0·179 | 1·128 |
|  | Rural*Low | 0·520 | 0·492 | 0·291 | 1·682 | 0·617 | 4·344 |
|  | Semi-Rural*Low | 0·539 | 0·319 | 0·091 | 1·714 | 0·938 | 3·296 |
|  | Urban*Low | -0·021 | 0·320 | 0·949 | 0·980 | 0·534 | 1·888 |
|  | Rural*Very Low | 0·872 | 0·426 | 0·041 | 2·392 | 1·032 | 5·547 |
|  | Semi-Rural*Very Low | 0·152 | 0·335 | 0·650 | 1·164 | 0·613 | 2·304 |
|  | Urban*Very Low | -0·198 | 0·337 | 0·556 | 0·820 | 0·430 | 1·626 |
|  | Rural*Extremely Low | 1·559 | 0·415 | <0·001 | 4·754 | 2·128 | 10·917 |
|  | Semi-Rural*Extremely Low | 0·512 | 0·389 | 0·189 | 1·668 | 0·779 | 3·616 |
|  | Urban*Extremely Low | Reference Category | | | | | |

^+^*coefficients and confidence intervals are not estimatable due to insufficient responses*
